# Comparing six antihypertensive medication classes for preventing new‐onset diabetes mellitus among hypertensive patients: a network meta‐analysis

**DOI:** 10.1111/jcmm.13096

**Published:** 2017-02-23

**Authors:** Yang Yang, Huilan Xu

**Affiliations:** ^1^ Department of Social Medicine School of Public Health Central South University Changsha Hunan China

**Keywords:** new‐onset diabetes, angiotensin II receptor blockers, angiotensin II converse enzyme inhibitors, calcium channel blockers, β‐blockers, diuretic, hypertension

## Abstract

Hypertensive patients usually have a higher risk of new‐onset diabetes mellitus (NOD) which may trigger cardiovascular diseases. In this study, the effectiveness of six antihypertensive agents with respect to NOD prevention in hypertensive patients was assessed. A network meta‐analysis was conducted to compare the efficacy of specific drug classes. PubMed and Embase databases were searched for relevant articles. Results of the pairwised meta‐analysis were illustrated by odd ratios (OR) and a corresponding 95% confidence interval (CI). The probabilities and outcome of each treatment were ranked and summarized using the surface under the cumulative ranking curve (SUCRA).Twenty‐three trials were identified, including 224,832 patients with an average follow‐up period of 3.9 ± 1.0 years. The network meta‐analysis showed that patients treated by angiotensin II receptor blockers (ARBs) were associated with a lower risk of NOD compared to placebo (PCB), calcium channel blockers (CCBs) and β‐blockers, while diuretic appeared to be ineffective for NOD prevention. Network meta‐analysis results of specific drugs showed that enalapril exhibited distinct advantages and hydrochlorothiazide also exhibited a reliable performance. Our results suggested that both ARBs and angiotensin converse enzyme inhibitors (ACEIs), especially candesartan and enalapril, were preferable for NOD prevention in hypertensive patients. Hydrochlorothiazide also exhibited a reliable performance in comparison with other agents.

## Introduction

NOD and hypertension often co‐existed, and thereby, the risk of cardiovascular diseases is substantially increased 1, 2. And previous evidence showed that this clinical dilemma was associated with an increased risk of hepatitis C virus infection and graft rejection and loss and thereby affected patient survival quality 3. Also, it was reported that diabetes may be prevented by renin–angiotensin blockers 4, 5. Recently, a number of trials on antihypertensive medications such as angiotensin‐converting enzyme inhibitors (ACEIs), ARBs, CCBs, diuretics and β‐blockers have explored whether these medications influenced NOD development 2, 6, 7.

Studies showed that ACEI or ARB could reduce the incidence of NOD in patients 8. ACEI therapy contributed to significant reduction compared with diuretics and β‐blockers 9, and it is potentially effective in reducing hypertension and cardiovascular risks 9. ARB is an antihypertensive agent which is able to reduce NOD development by enhancing the insulin sensitivity 10. However, it did not improve clinical outcome of cardiovascular diseases and no significant evidence was revealed from former trials 11.

Clinical trials reviewed that the use of valsartan in patients with cardiovascular disease resulted in a 14% reduction in NOD, while no significant therapeutic improvement for cardiovascular disease was confirmed 12. CCB could significantly reduce the incidence of NOD, and CCB combined with ARB had metabolically neutral effects 13. Effects of β‐blockers on NOD patient are controversial which might contribute to a reduced mortality and morbidity of heart failure among patients with NOD 14, while it might trigger the development of NOD 6. It was also indicated that the use of diuretic is associated with a decreased incidence of NOD 15 and prolonged diuretic treatment may result in an increased fasting glucose 15. In addition, the overall glycemic status was affected when both diuretics and β‐blockers are combined together 15. However, a study based on Indian population suggested that diuretics might increase the risk of NOD; that is, hypertensive patients treated with β‐blockers and diuretics exhibited higher incidence of diabetes mellitus 13.

Although previous meta‐analyses concluded that some antihypertensive medications were effective in NOD prevention, uncertain and controversy still remained to be clarified 4, 8, 9, 10, 16, 17, 18, 19, 20, 21, 22, 23, 24, besides, meta‐analyses were limited by few trials with direct comparisons between two treatments. Instead, a network meta‐analysis (NMA) can be conducted if both treatments have been compared to a common comparator. Formally, NMA can be defined as a statistical combination of all available evidence for an outcome from several studies across multiple treatments to generate estimates of pairwise comparison of each intervention to every other intervention within a network 25. As a result, we performed this NMA to compare the relative effectiveness of several antihypertensive medications including ACEIs (enalapril, lisinopril, perindopril, quinapril, ramipril, trandolapril), ARBs (include candesartan, losartan, telmisartan, valsartan), CCBs (amlodipine, verapamil), diuretics (bendrofluazide, chlorthalidone, hydrochlorothiazide) and β‐blockers (atenolol, propranolol).

## Materials and methods

### Data search

PubMed and Embase were searched, and studies from January 1985 up to June 2016 were identified to evaluate the efficacy of ACEIs, ARBs, CCBs, β‐blockers and diuretic. ARBs, ACEIs, CCBs, β‐blockers or diuretics and individual agent names within the medication classes were combined with ‘diabetes’, ‘pre‐diabetes’, ‘new‐onset diabetes mellitus’, ‘new‐onset diabetes’, ‘NOD’, ‘hypertension’, ‘high risk’, and ‘randomized, controlled, trials’’. Reference lists of identified articles including previous meta‐analyses and reviews were evaluated for additional relevant studies and information.

### Selection criteria

Studies were included if they fulfilled all the criteria as following: (*i*) comparison among ARBs, ACEIs, CCBs, β‐blockers, diuretics and PCB or other routine treatments; (*ii*) inclusion of individuals with hypertension or other high‐risk factors; (*iii*) the incidence of NOD as primary end‐point; (*iv*) average follow‐up over 1 year, recruiting more than 100 patients. Trials which did not meet above requirements were excluded.

### Data extraction and quality assessment

For each trial, all data derived from the published tables or texts were tabulated into a Microsoft Excel spreadsheet and the corresponding study characteristics were reviewed. In all data derived from each trial, we included total number of patients (overall population), number of patients with NOD at baseline, type and dosage of medications (ARBs, ACEIs, CCBs, β‐blockers, diuretics), follow‐up duration and other key study information.

### Statistical analysis

The incidence of NOD was treated as a dichotomous variable and assessed by odds ratios (ORs) with 95% confidential intervals (CIs) for six antihypertensive. Then, a subgroup analysis was conducted to compare the efficacy of specific drugs using NMA. Pooled ORs were calculated using the DerSimonian and Laird random‐effects model 26 or the Mantel–Haenszel fixed‐effects model 27, depending on the heterogeneity of treatment effects across studies. Bayesian statistical model was used. The percentage variability of the pooled ORs attributable to heterogeneity among the selected studies was quantified using the *I* ^2^ statistic test 28. Typically, values above 50% were deemed to suggest large between‐study heterogeneity. Under such a circumstance, the random‐effects model was used to improve accuracy of research. Ranking of medication with respect to the effectiveness of NOD prevention was achieved using the surface under the cumulative ranking area (SUCRA). A higher SUCRA value indicates a more desirable property with respect to a certain end‐point. Statistical analyses were conducted using R version 3.1.3 (R Project for Statistical Computing, Vienna, Austria). *P <* 0.05 was considered significantly different.

## Results

### Study selection

**Figure 1:**
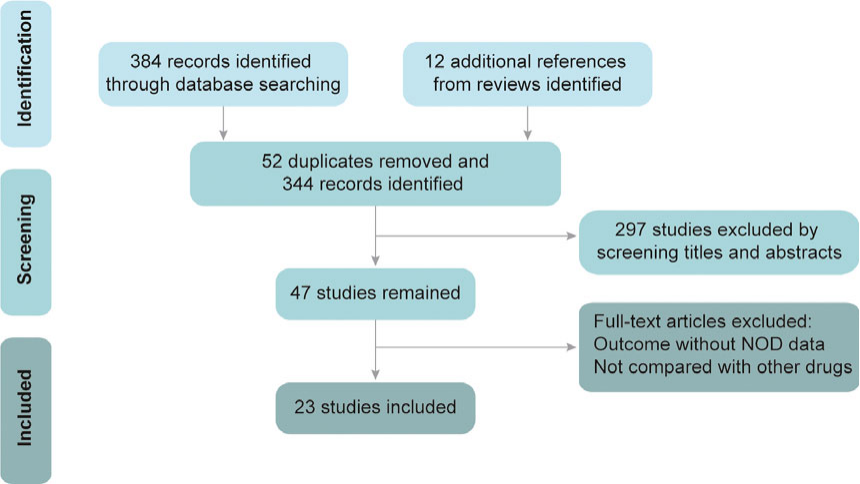
Study flow diagram. NOD: new‐onset diabetes.

As schematically shown in Figure 1, among the 396 potentially eligible trials, 52 duplicates were removed, 297 studies were excluded by screening titles and abstracts and 24 full‐text articles were ruled out as their outcome did not contain NOD data or medications were not properly compared. Thus, there were 23 totally randomized clinical trials, including a total of 224,832 patients, following as the selected criteria and selected in this meta‐analysis study 29, 30, 31, 32, 33, 34, 35, 36, 37, 38, 39, 40, 41, 42, 43, 44, 45, 46, 47, 48, 49, 50, 51.

### Population characteristics

**Figure 2:**
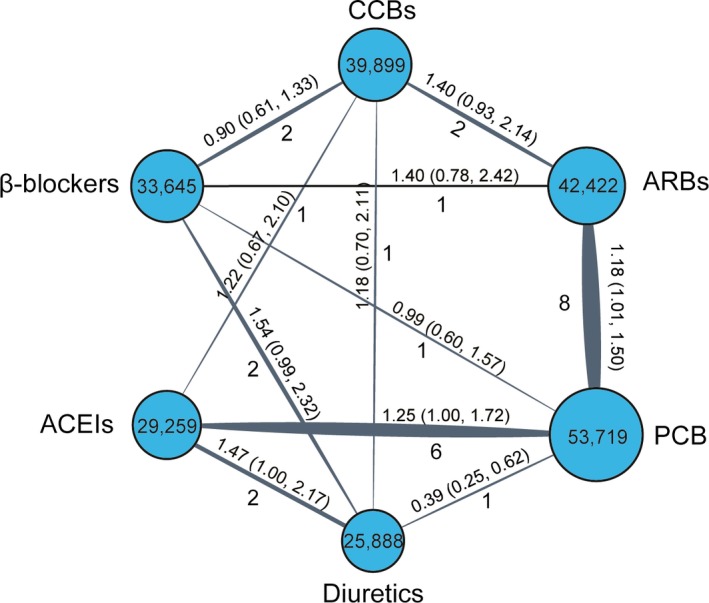
Network plot of eligible comparisons of categorized six different medications in NOD in the meta‐analysis. The width of the lines represents the total number of trials for each comparison. ACEIs: angiotensin converse enzyme inhibitors; ARBs: angiotensin II receptor blockers; CCBs: calcium channel blockers; NOD: new‐onset diabetes.

The general characteristics of the identified trials are shown in Table 1. Eight trials were designed to compare ARBs‐based treatments against PCB; six trials were aimed to compare ACEIs‐based treatments against PCB; and nine trials were designed to compare to each other among the five different treatments, namely ARBs, ACEIs, CCBs, β‐blockers or diuretic. A total of 224,832 hypertensive patients were involved in our study. A total of 53,719 (23.9%) patients were treated by PCB, 42,422 (18.9%) patients were randomized to receive ARBs, 39,899 (17.7%) patients received CCBs, 33,645 (15.0%) patients were treated by β‐blockers, 29,259 (13.0%) were treated by ACEIs and 25,888 (11.5%) were treated by diuretics. Network plots of six different kinds of medications and 18 agents were shown in Figure 2 and Figure S1. The mean age of these identified patients ranged from 51 to 72 years, and the duration was over an average follow‐up period of 3.9 ± 1.0 years.

**Table 1:** Characteristics of studies included in the network meta‐analysis

Study	Blinding	Duration (years)	Treatment class	Treatment drugs	Mean age	BP (mmHg)	Sample size
MRC trail,1985	Single‐blind	4.9	β‐Blocker/diuretic/PCB	Propranolol/bendrofluazide/Placebo	51/51/51	158/98	4403/4297/8654
Wilhelmsen,1987	–	3.8	β‐Blocker/diuretic	Atenolol/metoprolol/bendrofluazide/hydrochlorothiazide	52.3/52.2	166/107	3727/3297
Yusuf,2001	Double‐blind	4.5	ACEI/PCB	Ramipril/placebo	66.3/65.9	136.4/78.2	2837/2883
ALLHAT officers,2002	Double‐blind	4.9	ACEI/diuretic/CCB	Lisinopril/chlorthalidone/amlodipine	67/67/67	146/84	9054/15,255/9048
Lindholm,2002	Double‐blind	4.8	ARB/β‐blocker	Losartan/atenolol	66.9/66.9	174.3/97.9	4605/4588
Fox,2003	Double‐blind	4.3	ACEI/PCB	Perindopril/placebo	60/60	137/82	6110/6108
Vermes,2003	Double‐blind	2.9	ACEI/PCB	Enalapril/placebo	56.1/56.8	127.4/77.8	153/138
Wing,2003	–	4.1	ACEI/diuretic	Enalapril/hydrochlorothiazide	72/71.9	167/91	3044/3039
Pfeffer,2003	Double‐blind	3.1	ARB/PCB	Candesartan/placebo	65.9/66	130.6/76.6	3803/3796
Littell,2003	Double‐blind	3.7	ARB/PCB	Candesartan/placebo	76.4/76.4	166/90.3	2477/2460
Granger,2003	Double‐blind	2.8	ARB/PCB	Candesartan/placebo	66.3/66.8	127/78	1013/1015
Yusuf,2003	Double‐blind	3.1	ARB/PCB	Candesartan/placebo	67.2/67.1	130.6/76.6	1514/1509
Pepine,2003	–	2.7	CCB/β‐blocker	Verapamil/atenolol	66/66	149.5/86.3	11,267/11,309
McMurray,2003	Double‐blind	3.4	ARB/PCB	Candesartan/placebo	64/64.1	166/90.3	1276/1272
Braunwald,2004	Double‐blind	4.8	ACEI/PCB	Trandolapril/placebo	64/64	134/78	4158/4132
Julius,2004	Double‐blind	4.2	ARB/CCB	Valsartan/amlodipine	67.2/67.3	154.5/87.4	7649/7596
Dahlof,2005	Single‐blind	5.5	β‐Blocker/CCB	Atenolol/amlodipine	63/63	130/‐	9618/9639
Dream Investigators,2006	Double‐blind	3	ACEI/PCB	Ramipril/placebo	54.7/54.7	136.1/83.4	2623/2646
Yusuf,2008	–	2.5	ARB/PCB	Telmisartan/placebo	66.1/66.2	144.1/83.8	10,146/10,186
Ogihara,2008	–	3.2	ARB/CCB	Candesartan/amlodipine	63.8/63.9	162.5/91.6	2354/2349
TRANSCEND,2008	Double‐blind	4.7	ARB/PCB	Telmisartan/placebo	66.9/66.9	140.7/81.8	2954/2972
Rouleau,2008	Double‐blind	2.95	ACEI/PCB	Quinapril/placebo	61/61	122/70	1280/1273
NAVIGAROR group,2010	Double‐blind	6	ARB/PCB	Valsartan/placebo	63.7/63.8	139.4/82.5	4631/4675

BP: blood pressure; PCB: placebo; ACEI: angiotensin converse enzyme inhibitor; CCB: calcium channel blockers; ARB: angiotensin II receptor blockers.

### Incidence of NOD

**Figure 3:**
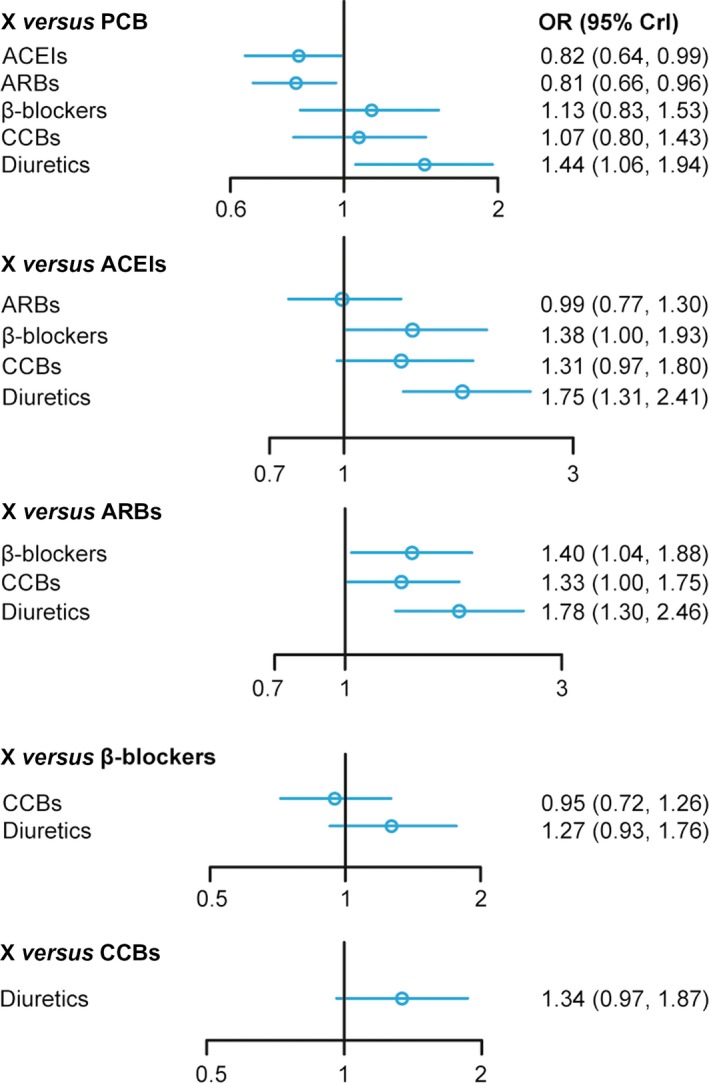
Forest plot for ARBs, ACEIs, CCBs, β‐blockers, diuretic or PCB‐based strategy on the incidence of NOD. ACEIs: angiotensin converse enzyme inhibitors; ARBs: angiotensin II receptor blockers; CCBs: calcium channel blockers; NOD: new‐onset diabetes.

As shown in Table 2, both ACEIs and ARBs showed a significant decline in the incidence of NOD compared to PCB, (OR = 0.82, 95% CrI = 0.64–0.99; OR = 0.81, 95% CrI = 0.66–0.96).And diuretics were associated with a higher risk of NOD compared with PCB (OR = 1.44 95% CrI = 1.06–1.94). Treatment of β‐blockers and diuretics showed a higher incidence of NOD than ACEIs (OR = 1.38, 95% CrI = 1.00–1.93; OR = 1.75, 95% CrI = 1.31–2.41), whereas β‐blockers, CCBs and diuretics also showed a significant elevation in the incidence of NOD compared to ARBs (OR = 1.40, 95% CrI = 1.04–1.88; OR = 1.33, 95% CrI = 1.00–1.75; OR = 1.78, 95% CrI = 1.30–2.46). Figure 3 illustrated the forest plot of network results.

**Table 2:** Results of six interventions for the incidence of new‐onset diabetes (NOD) from network meta‐analysis

Treatment	PCB	Diuretics	CCBs	β‐Blockers	ARBs	ACEIs
PCB	**PCB**	**1.44 (1.06, 1.94)**	1.07 (0.80, 1.43)	1.13 (0.83, 1.53)	**0.81 (0.67, 0.96)**	**0.82 (0.65, 1.00)**
Diuretics	**0.70 (0.52, 0.95)**	**Diuretics**	0.74 (0.54, 1.03)	0.79 (0.57, 1.07)	**0.56 (0.41, 0.77)**	**0.57 (0.42, 0.76)**
CCBs	0.94 (0.70, 1.26)	1.34 (0.97, 1.87)	**CCBs**	1.06 (0.80, 1.39)	**0.75 (0.57, 1.00)**	0.77 (0.56, 1.04)
β‐Blockers	0.89 (0.65, 1.21)	1.27 (0.93, 1.76)	0.94 (0.72, 1.26)	**β‐Blockers**	**0.71 (0.53, 0.96)**	**0.73 (0.52, 1.00)**
ARBs	**1.24 (1.04, 1.49)**	**1.78 (1.30, 2.46)**	**1.33 (1.00, 1.75)**	**1.40 (1.04, 1.88)**	**ARBs**	1.02 (0.77, 1.31)
ACEIs	**1.22 (1.00, 1.54)**	**1.75 (1.31, 2.41)**	1.31 (0.97, 1.80)	**1.38 (1.00, 1.93)**	0.98 (0.76, 1.30)	**ACEIs**

Bold values mean statistic difference.

### Ranking of antihypertensive medications by SUCRA

**Figure 4:**
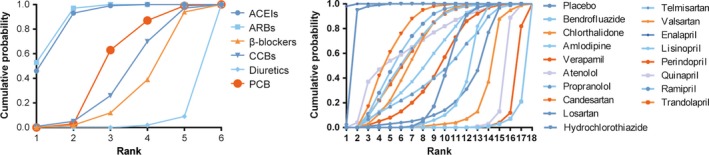
Rank graphs showing the probability of six different kinds of medications having each specific rank (1–6) and the probability of three kinds of ARBs having each specific rank (1–4) for end‐points. Ranking suggests the probability to be the best intervention treatment, the second best and so on. Rank 1st is best and Rank 6th is worst.

The probability of six antihypertensive medications having specific rank (1–6) and the probability of three kinds of ARBs having each specific rank (1–4) for the incidence of NOD are presented in Figure 4. SUCRA showed that both ARBs (SUCRA = 0.894) and ACEIs (SUCRA = 0.880) exhibited distinct advantages compared to the other four treatments and diuretics (SUCRA = 0.022) exhibited the last least reliable performance in comparison with other medications. Candesartan was considered to be more desirable than other ARBs (Table 3 and Table S1).

**Table 3:** Surface under the cumulative ranking curve (SUCRA) of 18 treatments according to NOD

Classification	Drugs	SUCRA	Mean rank
ACEI	Enalapril	0.998	1
ACEI	Ramipril	0.700	4
ACEI	Quinapril	0.698	5
ACEI	Trandolapril	0.661	7
ACEI	Lisinopril	0.558	9
ACEI	Perindopril	0.485	10
ARB	Candesartan	0.735	3
ARB	Telmisartan	0.675	6
ARB	Valsartan	0.628	8
ARB	Losartan	0.306	14
β‐Blocker	Propranolol	0.475	11
β‐Blocker	Atenolol	0.122	16
CCB	Amlodipine	0.326	13
CCB	Verapamil	0.059	17
Diuretic	Hydrochlorothiazide	0.938	2
Diuretic	Chlorthalidone	0.203	15
Diuretic	Bendrofluazide	0.020	18
Placebo	Placebo	0.424	12

### Assessing inconsistency between direct and indirect evidence

**Figure 5:**
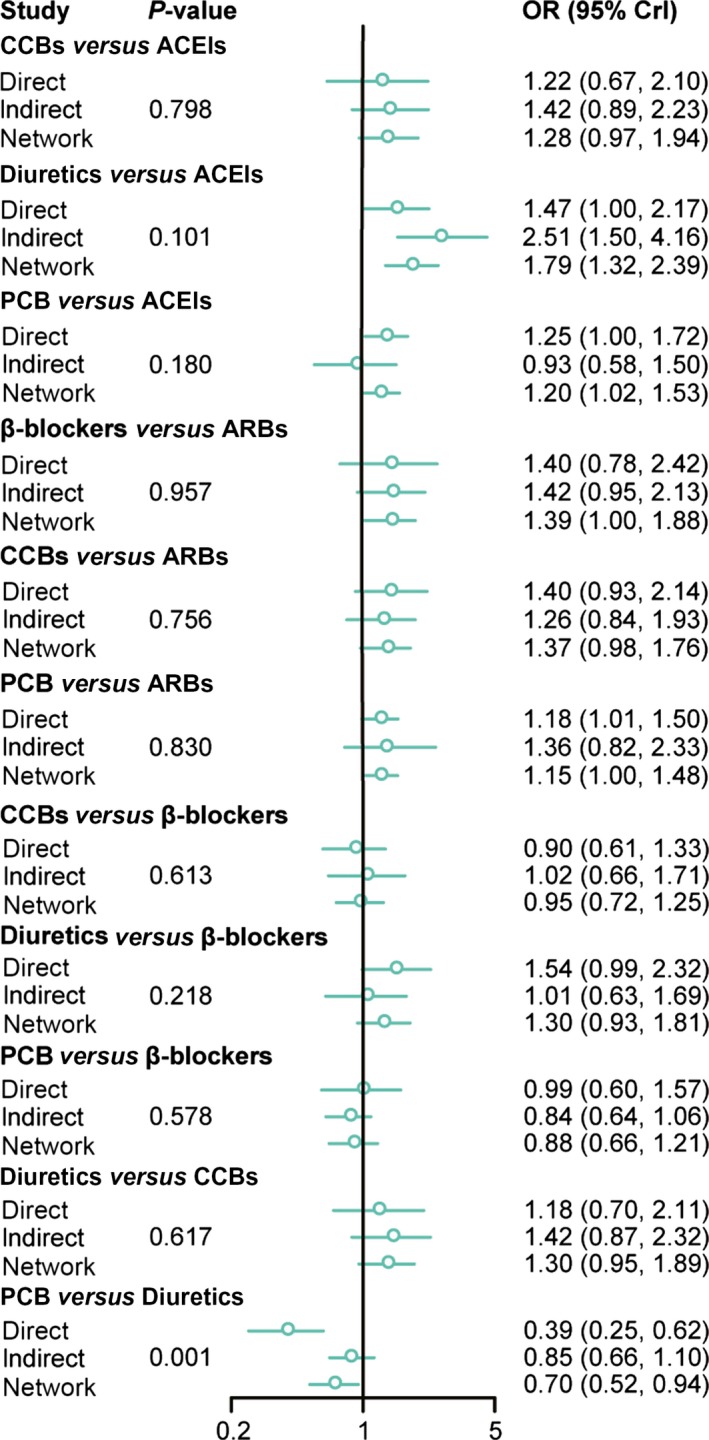
Summarized results of direct and indirect comparisons between ARBs, ACEIs, CCBs, β‐blockers, diuretic or PCB‐based strategy on the incidence of NOD. ACEIs: angiotensin converse enzyme inhibitors; ARBs: angiotensin II receptor blockers; CCBs: calcium channel blockers; NOD: new‐onset diabetes.

One fundamental assumption in our NMA is the adoption of a consistency model in which the extent of consistency is validated using the node splitting method. Results of direct, indirect and network comparisons of these interventions were displayed in node splitting forest plots as shown in Figure 5 and a *P*‐value of less than 0.05 suggests potentially significant inconsistency and hence, the consistency model assumption may be violated. The inconsistency only exists in comparison between PCB and diuretics (*P*‐value = 0.001). As it is the only case in which potential significant inconsistency may arise from, we did not replace the consistency model in our NMA.

## Discussion

Patients with hypertension usually have a higher risk of NOD which may trigger cardiovascular diseases 52. Preventing NOD among patients with hypertension has been considered as a prioritized task by clinicians. Current antihypertensive medications are generally divided into several classes, namely thiazide diuretic, ACEIs, ARBs, CCBs and β‐blockers 53. In this study, we collected data from 23 NOD studies which investigated six antihypertensive medications in order to assess their efficacy with respect to NOD prevention. We aimed to provide conclusive evidence for ranking these medications so that potential guidance with respect to medication selection can be recommended to clinicians.

In this study, the results of NMA showed that patients treated by ARBs or ACEIs were associated with a reduced risk of NOD compared to those with PCB, while diuretic appeared to be ineffective with respect to NOD prevention. ARBs also exhibited a better performance with respect to NOD prevention compared to CCBs or β‐blockers. As suggested by the overall rank, both ARBs and ACEIs, especially enalapril and candesartan, were more preferable than other treatments and hydrochlorothiazide also exhibited a reliable performance in comparison with other agents.

Previous studies demonstrated that the renin–angiotensin system was activated in all insulin resistant states in which type II diabetes or hypertension may be involved 54. Blocking the RAS not only improved blood circulation and cellular ionic balance of pancreatic and skeletal muscle cells, but also enhanced the effects of peripheral insulin and insulin secretion by promoting the recruitment and differentiation of adipocytes in diabetes 55. Recent studies showed that hypertensive patients treated by ACEIs or ARB were associated with a lower risk of NOD or adverse cardiovascular events 56, 57, 58 and such a mechanism may involve the improvements in both insulin sensitivity and secretion 59. However, ARBs and ACEIs have different mechanisms with respect to preventing insulin resistance. For instance, ACEIs inhibits the conversion from Ang I to Ang II and blocks the degradation of bradykinin whereas ARBs suppresses Ang II by selectively binding to the corresponding receptor site 60. In this study, both direct and indirect evidence confirmed that patients treated by ARBs or ACEIs were associated with a reduced risk of NOD compared to those with PCB, while diuretics appeared to be ineffective with respect to NOD prevention and ARBs also exhibited better performance with respect to NOD prevention compared to CCBs or β‐blockers. Compared to diuretics, ACEIs is potentially more cost‐effective for elderly hypertensive patients 1. The corresponding mechanism of ACEIs may be linked with the lack of major sympathoexcitatory effects improvements in insulin sensitivity 61.

Diuretics have been widely used for managing salt‐sensitive hypertension, and they are divided into diuretics, such as hydrochlorothiazide and thiazide‐like diuretics such as chlorthalidone and bendrofluazide 62. A recent study showed that hydrochlorothiazide was inferior to indapamide for improving endothelial functions and ventriculoarterial coupling in patients who suffered from both hypertension and diabetes 63. In this study, patients treated by ARBs or ACEIs were associated with a significant reduction in the incidence of NOD compared to those treated by bendrofluazide or chlorthalidone. However, patients treated by hydrochlorothiazide exhibited a significant reduction in the risk of NOD compared to those treated by ARBs, ACEIs, CCBs, β‐blockers or other diuretics except enalapril. CCBs and β‐blockers, as two other first‐line antihypertensive medications, are effective in preventing cardiovascular events 64 and are generally prescribed by clinicians as hypertension therapies 65. It has been shown that CCBs or β‐blockers had mild or no impact on the risk of NOD 66. Results from our study indicated that patients treated by CCBs or β‐blockers seemed to have equivalent risk of NOD.

As the first Bayesian NMA, our study compared six antihypertensive medications that were used in hypertensive patients for preventing NOD and the corresponding data were synthesized from the current literature. However, a few limitations contained in this study should be concerned and addressed in the future. Firstly, there was significant variation in the number of studies with respect to each comparison. For instance, the number of studies which compared losartan, propranolol, chlorthalidone or bendrofluazide was significantly less than that of others and this may result in wide confidence interval for summary statistics when data were synthesized. Secondly, variation in the sample size and study duration within each individual study as well as variation in other study characteristics may cause significant heterogeneity and thereby pooling evidence from individual studies with significant heterogeneity may not be comparable.

For summary, both ARBs and ACEIs exhibited compelling results with respect to the prevention of NOD and such a trend is even more significant in patients treated by enalapril (ACEIs) or candesartan (ARBs). Apart from that, hydrochlorothiazide also exhibited reliable performance in comparison with other agents. Large‐scale randomized trials should be designed and implemented to confirm the above conclusions.

## Disclosure of interest

The authors declare that they have no conflict of interests concerning this article.

## Supporting information


**Figure S1.** Network plot of eligible studies comparing 18 agents included in six different kinds of medications in NOD. The width of the lines represents the total number of trials for each comparison.

**Table S1**. Results of 17 antihypertensive agents and placebo for the incidence of new onset diabetes (NOD) from net‐work meta‐analysis.
