# Supplementary figures and images for: Comparing six antihypertensive medication classes for preventing new‐onset diabetes mellitus among hypertensive patients: a network meta‐analysis
Source: J Cell Mol Med. 2017 Feb 23;21(9):1742–50. doi: 10.1111/jcmm.13096 (PMC5571556; doi:10.1111/jcmm.13096)

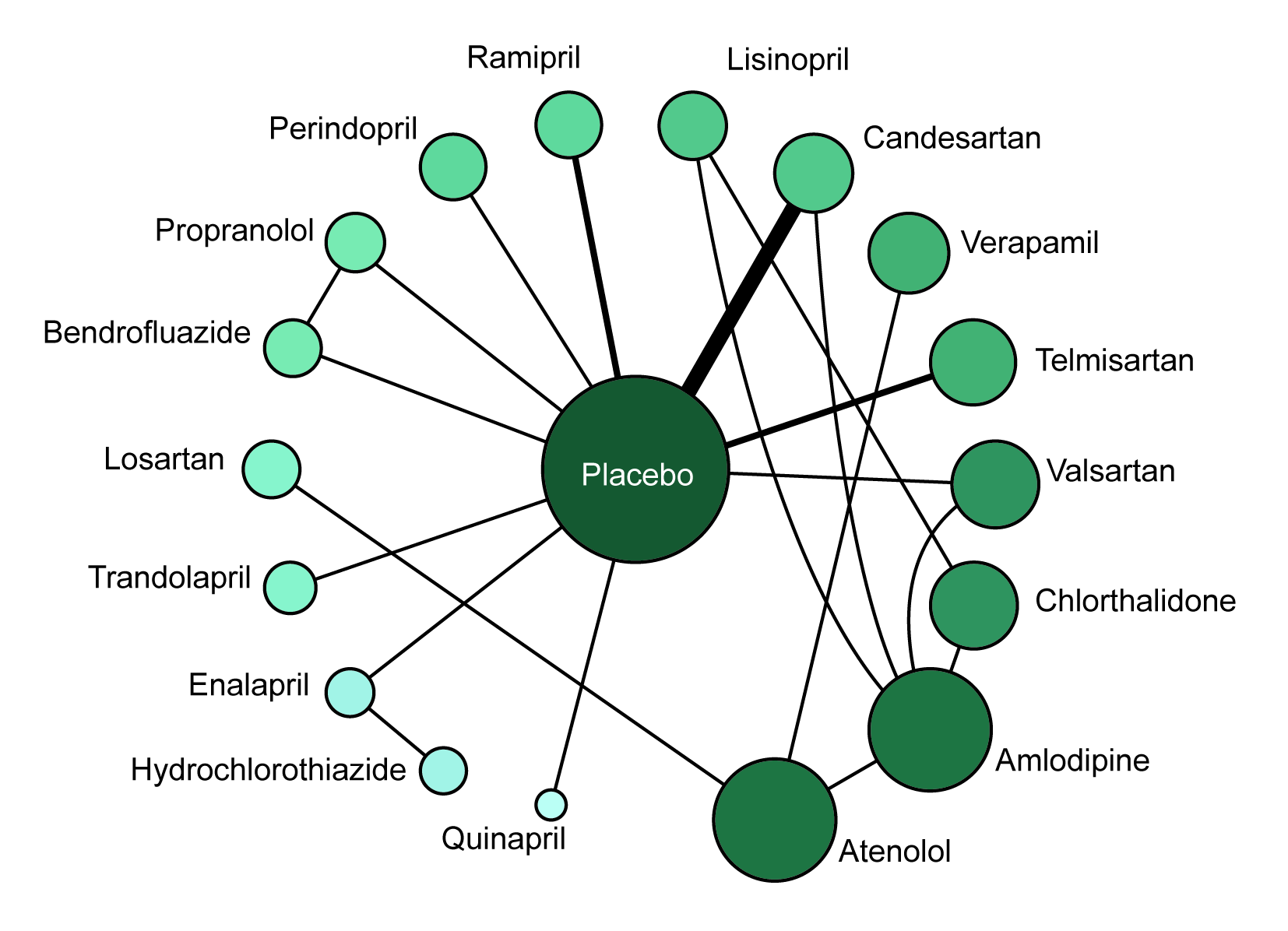

Supplement: Supplementary file 1 — Figure S1. Network plot of eligible studies comparing 18 agents included in six different kinds of medications in NOD. The width of the lines represents the total number of trials for each comparison. [file JCMM-21-1742-s001.tif]
