# Supplementary material for: Comparing six antihypertensive medication classes for preventing new‐onset diabetes mellitus among hypertensive patients: a network meta‐analysis
Source: J Cell Mol Med. 2017 Feb 23;21(9):1742–50. doi: 10.1111/jcmm.13096 (PMC5571556; doi:10.1111/jcmm.13096)
Supplement: Supplementary file 2 — Table S1. Results of 17 antihypertensive agents and placebo for the incidence of new onset diabetes (NOD) from net‐work meta‐analysis. [file JCMM-21-1742-s002.docx]

**Table S1. Results of seventeen antihypertensive agents and placebo for the incidence of new onset diabetes (NOD) from net-work meta-analysis**

| **Treatment** | **Placebo** | **Bendrofluazide** | **Chlorthalidone** | **Hydrochlorothiazide** | **Amlodipine** | **Verapamil** | **Atenolol** | **Propranolol** | **Candesartan** | **Losartan** | **Telmisartan** | **Valsartan** | **Enalapril** | **Lisinopril** | **Perindopril** | **Quinapril** | **Ramipril** | **Trandolapril** |
| --- | --- | --- | --- | --- | --- | --- | --- | --- | --- | --- | --- | --- | --- | --- | --- | --- | --- | --- |
| **Placebo** | **Placebo** | **2.55 (1.70, 3.84)** | 1.37 (0.87, 2.24) | **0.30 (0.11, 0.77)** | 1.14 (0.82, 1.63) | **1.97 (1.08, 3.51)** | **1.67 (1.06, 2.73)** | 0.99 (0.61, 1.58) | **0.78 (0.66, 0.95)** | 1.22 (0.70, 2.26) | 0.83 (0.63, 1.08) | 0.86 (0.65, 1.16) | **0.20 (0.08, 0.49)** | 0.92 (0.58, 1.50) | 0.96 (0.68, 1.36) | 0.80 (0.45, 1.41) | 0.81 (0.61, 1.03) | 0.84 (0.59, 1.19) |
| **Bendrofluazide** | **0.39 (0.26, 0.59)** | **Bendrofluazide** | 0.54 (0.29, 1.04) | **0.12 (0.04, 0.33)** | **0.45 (0.26, 0.77)** | 0.77 (0.37, 1.58) | 0.66 (0.35, 1.25) | **0.39 (0.24, 0.62)** | **0.31 (0.20, 0.48)** | **0.48 (0.24, 0.99)** | **0.32 (0.20, 0.54)** | **0.34 (0.20, 0.57)** | **0.08 (0.03, 0.21)** | **0.36 (0.20, 0.69)** | **0.38 (0.22, 0.64)** | **0.31 (0.15, 0.65)** | **0.32 (0.19, 0.51)** | **0.33 (0.19, 0.56)** |
| **Chlorthalidone** | 0.73 (0.45, 1.15) | 1.86 (0.96, 3.42) | **Chlorthalidone** | **0.21 (0.07, 0.61)** | 0.83 (0.60, 1.15) | 1.44 (0.78, 2.53) | 1.22 (0.77, 1.90) | 0.72 (0.36, 1.39) | **0.57 (0.35, 0.90)** | 0.89 (0.49, 1.60) | 0.61 (0.35, 1.02) | **0.63 (0.40, 0.96)** | **0.15 (0.05, 0.39)** | **0.67 (0.48, 0.93)** | 0.71 (0.38, 1.24) | 0.58 (0.27, 1.21) | **0.59 (0.33, 0.97)** | 0.61 (0.33, 1.04) |
| **Hydrochlorothiazide** | **3.37 (1.30, 8.92)** | **8.68 (3.05, 25.20)** | **4.67 (1.63, 13.77)** | **Hydrochlorothiazide** | **3.85 (1.42, 10.98)** | **6.65 (2.17, 20.92)** | **5.64 (2.01, 17.23)** | **3.33 (1.13, 10.31)** | **2.64 (1.01, 7.17)** | **4.13 (1.42, 13.28)** | **2.81 (1.03, 7.68)** | **2.91 (1.07, 8.10)** | **0.68 (0.47, 0.99)** | **3.13 (1.08, 9.30)** | **3.28 (1.18, 9.07)** | 2.67 (0.89, 8.18) | **2.73 (1.01, 7.59)** | **2.84 (1.03, 7.98)** |
| **Amlodipine** | 0.88 (0.62, 1.22) | **2.24 (1.29, 3.82)** | 1.21 (0.87, 1.67) | **0.26 (0.09, 0.70)** | **Amlodipine** | **1.74 (1.04, 2.78)** | **1.47 (1.05, 2.06)** | 0.87 (0.48, 1.53) | **0.69 (0.48, 0.96)** | 1.07 (0.67, 1.78) | 0.73 (0.47, 1.11) | 0.75 (0.56, 1.01) | **0.18 (0.07, 0.45)** | 0.81 (0.58, 1.13) | 0.85 (0.50, 1.35) | 0.70 (0.34, 1.35) | 0.71 (0.44, 1.04) | 0.73 (0.44, 1.17) |
| **Verapamil** | **0.51 (0.28, 0.93)** | 1.29 (0.63, 2.71) | 0.69 (0.40, 1.28) | **0.15 (0.05, 0.46)** | **0.58 (0.36, 0.96)** | **Verapamil** | 0.85 (0.61, 1.22) | 0.50 (0.24, 1.09) | **0.40 (0.23, 0.73)** | 0.62 (0.39, 1.05) | **0.42 (0.22, 0.81)** | 0.44 (0.25, 0.78) | **0.10 (0.03, 0.29)** | **0.47 (0.27, 0.86)** | **0.49 (0.25, 0.98)** | **0.41 (0.17, 0.92)** | **0.41 (0.21, 0.77)** | **0.42 (0.21, 0.83)** |
| **Atenolol** | **0.60 (0.37, 0.94)** | 1.52 (0.80, 2.86) | 0.82 (0.53, 1.30) | **0.18 (0.06, 0.50)** | **0.68 (0.49, 0.95)** | 1.18 (0.82, 1.63) | **Atenolol** | 0.59 (0.30, 1.14) | **0.47 (0.29, 0.75)** | 0.73 (0.52, 1.05) | **0.50 (0.28, 0.84)** | **0.51 (0.33, 0.79)** | **0.12 (0.04, 0.32)** | **0.55 (0.35, 0.87)** | 0.58 (0.31, 1.01) | **0.48 (0.22, 0.98)** | **0.49 (0.27, 0.79)** | **0.50 (0.28, 0.88)** |
| **Propranolol** | 1.01 (0.63, 1.64) | **2.58 (1.61, 4.16)** | 1.39 (0.72, 2.76) | **0.30 (0.10, 0.88)** | 1.15 (0.65, 2.07) | 1.98 (0.92, 4.16) | 1.69 (0.88, 3.33) | **Propranolol** | 0.79 (0.47, 1.34) | 1.24 (0.59, 2.68) | 0.84 (0.49, 1.44) | 0.87 (0.50, 1.52) | **0.21 (0.07, 0.56)** | 0.93 (0.48, 1.82) | 0.98 (0.54, 1.72) | 0.80 (0.37, 1.73) | 0.82 (0.47, 1.38) | 0.84 (0.47, 1.51) |
| **Candesartan** | **1.28 (1.06, 1.53)** | **3.25 (2.07, 5.09)** | **1.75 (1.11, 2.87)** | **0.38 (0.14, 0.99)** | **1.45 (1.04, 2.06)** | **2.52 (1.37, 4.44)** | **2.13 (1.34, 3.46)** | 1.26 (0.75, 2.12) | **Candesartan** | 1.56 (0.89, 2.88) | 1.05 (0.76, 1.47) | 1.10 (0.79, 1.52) | **0.26 (0.10, 0.63)** | 1.18 (0.75, 1.91) | 1.24 (0.82, 1.81) | 1.02 (0.55, 1.86) | 1.03 (0.73, 1.38) | 1.07 (0.72, 1.58) |
| **Losartan** | 0.82 (0.44, 1.43) | **2.07 (1.01, 4.21)** | 1.12 (0.62, 2.03) | **0.24 (0.08, 0.71)** | 0.93 (0.56, 1.50) | 1.61 (0.96, 2.58) | 1.36 (0.95, 1.93) | 0.81 (0.37, 1.71) | 0.64 (0.35, 1.12) | **Losartan** | 0.68 (0.35, 1.25) | 0.71 (0.40, 1.20) | **0.17 (0.05, 0.45)** | 0.75 (0.42, 1.33) | 0.79 (0.39, 1.52) | 0.65 (0.27, 1.46) | 0.66 (0.34, 1.18) | 0.69 (0.34, 1.29) |
| **Telmisartan** | 1.21 (0.92, 1.58) | **3.08 (1.86, 4.99)** | 1.65 (0.98, 2.87) | **0.36 (0.13, 0.98)** | 1.37 (0.90, 2.12) | **2.39 (1.23, 4.54)** | **2.02 (1.19, 3.52)** | 1.19 (0.70, 2.05) | 0.95 (0.68, 1.31) | 1.48 (0.80, 2.88) | **Telmisartan** | 1.03 (0.71, 1.54) | **0.25 (0.09, 0.61)** | 1.11 (0.66, 1.95) | 1.16 (0.74, 1.81) | 0.95 (0.50, 1.82) | 0.98 (0.66, 1.39) | 1.01 (0.66, 1.55) |
| **Valsartan** | 1.16 (0.86, 1.54) | **2.96 (1.76, 4.90)** | **1.60 (1.04, 2.52)** | **0.34 (0.12, 0.93)** | 1.33 (0.99, 1.79) | **2.30 (1.29, 4.01)** | **1.95 (1.26, 3.04)** | 1.15 (0.66, 1.99) | 0.91 (0.66, 1.26) | 1.42 (0.83, 2.52) | 0.97 (0.65, 1.42) | **Valsartan** | **0.24 (0.09, 0.59)** | 1.07 (0.70, 1.70) | 1.12 (0.71, 1.73) | 0.92 (0.47, 1.75) | 0.94 (0.62, 1.35) | 0.97 (0.62, 1.51) |
| **Enalapril** | **4.93 (2.03, 12.43)** | **12.61 (4.77, 34.13)** | **6.85 (2.58, 19.08)** | **1.48 (1.01, 2.15)** | **5.63 (2.22, 15.04)** | **9.69 (3.44, 29.06)** | **8.21 (3.11, 23.29)** | **4.87 (1.78, 14.04)** | **3.83 (1.58, 9.94)** | **6.01 (2.20, 18.33)** | **4.08 (1.63, 10.59)** | **4.25 (1.68, 11.20)** | **Enalapril** | **4.58 (1.73, 12.83)** | **4.75 (1.92, 12.40)** | **3.88 (1.39, 11.27)** | **3.95 (1.60, 10.46)** | **4.13 (1.64, 11.14)** |
| **Lisinopril** | 1.09 (0.67, 1.72) | **2.78 (1.45, 5.11)** | **1.49 (1.08, 2.07)** | **0.32 (0.11, 0.92)** | 1.23 (0.89, 1.72) | **2.14 (1.17, 3.77)** | **1.82 (1.15, 2.88)** | 1.08 (0.55, 2.06) | 0.85 (0.52, 1.33) | 1.33 (0.75, 2.40) | 0.90 (0.51, 1.51) | 0.93 (0.59, 1.44) | **0.22 (0.08, 0.58)** | **Lisinopril** | 1.05 (0.56, 1.86) | 0.87 (0.40, 1.79) | 0.88 (0.49, 1.45) | 0.91 (0.49, 1.60) |
| **Perindopril** | 1.04 (0.74, 1.47) | **2.64 (1.55, 4.60)** | 1.42 (0.81, 2.64) | **0.31 (0.11, 0.84)** | 1.18 (0.74, 1.98) | **2.03 (1.02, 4.06)** | 1.73 (0.99, 3.20) | 1.02 (0.58, 1.85) | 0.81 (0.55, 1.21) | 1.27 (0.66, 2.55) | 0.86 (0.55, 1.35) | 0.89 (0.58, 1.42) | **0.21 (0.08, 0.52)** | 0.95 (0.54, 1.77) | **Perindopril** | 0.82 (0.42, 1.63) | 0.84 (0.54, 1.27) | 0.86 (0.53, 1.41) |
| **Quinapril** | 1.25 (0.71, 2.24) | **3.21 (1.54, 6.71)** | 1.73 (0.83, 3.76) | 0.38 (0.12, 1.13) | 1.43 (0.74, 2.90) | **2.46 (1.08, 5.76)** | **2.10 (1.02, 4.57)** | 1.25 (0.58, 2.69) | 0.98 (0.54, 1.83) | 1.54 (0.68, 3.68) | 1.05 (0.55, 1.99) | 1.08 (0.57, 2.13) | **0.26 (0.09, 0.72)** | 1.16 (0.56, 2.52) | 1.21 (0.61, 2.38) | **Quinapril** | 1.01 (0.54, 1.91) | 1.05 (0.54, 2.07) |
| **Ramipril** | 1.24 (0.97, 1.64) | **3.16 (1.97, 5.26)** | **1.69 (1.03, 3.04)** | **0.37 (0.13, 0.99)** | 1.40 (0.96, 2.25) | **2.44 (1.31, 4.75)** | **2.06 (1.26, 3.75)** | 1.23 (0.72, 2.13) | 0.97 (0.73, 1.37) | 1.51 (0.85, 2.98) | 1.02 (0.72, 1.52) | 1.06 (0.74, 1.60) | **0.25 (0.10, 0.63)** | 1.13 (0.69, 2.04) | 1.20 (0.79, 1.87) | 0.99 (0.52, 1.86) | **Ramipril** | 1.03 (0.69, 1.61) |
| **Trandolapril** | 1.20 (0.84, 1.69) | **3.03 (1.80, 5.26)** | 1.64 (0.96, 3.03) | **0.35 (0.13, 0.97)** | 1.36 (0.86, 2.25) | **2.36 (1.21, 4.71)** | **2.00 (1.14, 3.63)** | 1.18 (0.66, 2.13) | 0.94 (0.63, 1.39) | 1.46 (0.78, 2.96) | 0.99 (0.65, 1.53) | 1.03 (0.66, 1.61) | **0.24 (0.09, 0.61)** | 1.10 (0.63, 2.05) | 1.16 (0.71, 1.89) | 0.95 (0.48, 1.86) | 0.97 (0.62, 1.46) | **Trandolapril** |
